# Pulse Wave Velocity as Marker of Preclinical Arterial Disease: Reference Levels in a Uruguayan Population Considering Wave Detection Algorithms, Path Lengths, Aging, and Blood Pressure

**DOI:** 10.1155/2012/169359

**Published:** 2012-05-16

**Authors:** Ignacio Farro, Daniel Bia, Yanina Zócalo, Juan Torrado, Federico Farro, Lucía Florio, Alicia Olascoaga, Walter Alallón, Ricardo Lluberas, Ricardo L. Armentano

**Affiliations:** ^1^Physiology Department, School of Medicine, CUiiDARTE, Republic University, General Flores 2125, 11800 Montevideo, Uruguay; ^2^Cardiology Department, School of Medicine, CUiiDARTE and Cardiovascular Centre, Clinical Hospital, Republic University, Avenida Italia s/n, 11600 Montevideo, Uruguay; ^3^Clinical Laboratory Department, School of Medicine, Clinical Hospital, Republic University, Avenida Italia s/n, 11600 Montevideo, Uruguay

## Abstract

Carotid-femoral pulse wave velocity (PWV) has emerged as the gold standard for non-invasive evaluation of aortic stiffness; absence of standardized methodologies of study and lack of normal and reference values have limited a wider clinical implementation. This work was carried out in a Uruguayan (South American) population in order to characterize normal, reference, and threshold levels of PWV considering normal age-related changes in PWV and the prevailing blood pressure level during the study. A conservative approach was used, and we excluded symptomatic subjects; subjects with history of cardiovascular (CV) disease, diabetes mellitus or renal failure; subjects with traditional CV risk factors (other than age and gender); asymptomatic subjects with atherosclerotic plaques in carotid arteries; patients taking anti-hypertensives or lipid-lowering medications. The included subjects (*n* = 429) were categorized according to the age decade and the blood pressure levels (at study time). All subjects represented the “reference population”; the group of subjects with optimal/normal blood pressures levels at study time represented the “normal population.” *Results*. Normal and reference PWV levels were obtained. Differences in PWV levels and aging-associated changes were obtained. The obtained data could be used to define vascular aging and abnormal or disease-related arterial changes.

## 1. Introduction

Noninvasive assessment of arterial stiffness has been proposed for individual cardiovascular risk evaluation and early detection of vascular damage associated with hypertension and/or atherosclerosis [1–4]. Among the different noninvasive methods used to assess arterial stiffness the carotid-femoral pulse wave velocity (PWV) has emerged as a *gold standard* due to its accuracy, reproducibility, relative easy measurement, and low costs [3, 4]. Furthermore, PWV has yielded prognostic value beyond and above traditional risk factors [3, 4]. However, and in spite of its recognized value the evaluation of PWV in the clinical practice has been hampered among other factors by the absence of standardized methodologies of study and the lack of established normal/reference values for different populations [3–8].

PWV assessment involves measuring the pulse wave transit time along the analyzed arterial segment and the distance on the skin between the pulse wave recording sites. Therefore, PWV values depend on both the path length measurement and the algorithm used to detect “the foot” of the analyzed waves [4]. The algorithms most frequently used are the intersecting tangent algorithm and the point of maximal upstroke during systole [3, 4]. The pathway can be the direct distance measured between the carotid and femoral measurement sites, the sternal notch-femoral measurement site distance, or the distance obtained by subtracting the carotid-sternal notch distance from the sternal notch-femoral distance (subtracted distance). Different algorithms applied to the same waves can lead to differences in PWV of 5–15%, while differences in path length alone can result in differences in PWV of up to 30% [3, 4]. When determining normal and/or reference values those technicality-related issues should be considered. Arterial stiffness also depends on blood pressure levels and increases with age [3, 4]. These latter are major determinants of PWV and despite several factors have shown to influence PWV, in many cases their effects would be negligible after correction for age [3].

Reference values for PWV have been recently provided from data mainly derived from European and/or North American populations [3]. Given the ethnic diversity in cardiovascular risk profile, the dissimilar risk associations, and the differences in genetic-environmental interactions among populations, studies performed in a given population could not be directly applied to another. Latin America encompasses a wide variety of geographic, ethnic, and socioeconomic differences. Such diversity would be reflected in the prevalence/profile of cardiovascular risk factors, atherosclerotic vascular changes, and/or in the reference values for the vascular parameters [9–12]. For instance, the CARMELA study showed large differences in the vascular characteristics (i.e., carotid intima-media thickness) among Latin American populations [12]. Then, considering the value of the vascular evaluation in cardiovascular risk stratification and diagnosis it is necessary to determine normal/reference levels for different vascular parameters taking into account the differences among populations. 

The present study (from CUiiDARTE Project) was carried out in a Uruguayan population to (a) quantify PWV normal/reference levels, (b) evaluate age-associated changes in PWV, and (c) analyze and compare the differences in PWV obtained using different wave detection algorithms and path lengths, considering age and pressure levels.

## 2. Materials and Methods

### 2.1. Study Population and Subjects Groups

CUiiDARTE Project is a population-based national study developed in Uruguay [13, 14]. With an area of approximately 176,000 square kilometers, Uruguay has a population of approximately 3.5 million (1.8 million live in Montevideo and its metropolitan area). Most Uruguayans (88%) are Caucasian of European origin (mainly Spanish).

#### 2.1.1. Subjects' Selection and Groups

Our study was designed to characterize the physiological age-associated changes in arterial parameters considered markers of vascular disease. Only knowing the physiological changes associated with age it would be possible to know whether a particular value of a vascular parameter is the result of normal aging or reflects a diseased arterial system.

Subjects referred for cardiovascular evaluation in CUiiDARTE Project were considered eligible. A probability sampling strategy (Cluster Sampling) was employed. We aimed at analyzing aging-related vascular changes. Then, a conservative approach was used, and we excluded subjects with cardiovascular symptoms, history of cardiovascular disease, diabetes mellitus or renal failure, cardiovascular risk factors other than age and gender (i.e., smokers), atherosclerotic plaques in carotid arteries (B-mode and Doppler ultrasound evaluation), and/or taking antihypertensives or lipid-lowering medications [13, 14]. To ensure an adequate application of the exclusion factors, we used a medical interview in which personal and family history and lifestyle habits were assessed (standardized questionnaire). Anthropometric and laboratory data were obtained (see below). The study was approved by the Institutional Ethic Committee, and it was conducted according to the Declaration of Helsinki and the Good Clinical Practice Guidelines. CUiiDARTE Centre and Project details can be found in http://www.cuiidarte.fmed.edu.uy/.

Subjects were studied in a single visit. Evaluation started after 9–12-hour overnight fast. Exercise, caffeine, alcohol, and vitamin C were avoided prior (at least six hours) to the cardiovascular examination. Subjects' height and weight were measured, and the body mass index (BMI, weight to height squared ratio) calculated. Subjects with a BMI higher than 30 Kg/m^2^ were excluded.

### 2.2. Laboratory Measurements

Venous blood samples were drawn and processed immediately using commercially available kits and/or laboratory methods. Procedures were standardized before the study initiation and during the study's development they were controlled for quality by a central reference laboratory (Clinical Laboratory Department, Clinical Hospital, School of Medicine). Total cholesterol (TC) was determined by the spectrophotometry cholesterol oxidase/peroxidase enzymatic method; serum triglycerides (TG) and high-density lipoprotein cholesterol (HDL-C) were determined by glycerol enzymatic method and the precipitating reactive method, respectively; low-density lipoprotein cholesterol (LDL-C) was calculated by the Friedewald formula (LDL-C = [TC − HDL-C] − [TG/5], valid if TG < 400 mg/dL) [15]. Non-HDL-C (TC − HDL-C) and TC/HDL-C were calculated.

Lipid values were classified according to NCEP-ATP III criteria [16]. Patients with a lipid profile with one or more of the following conditions: TG ≥ 200 mg/dL, TC ≥ 240 mg/dL, HDL-C < 40 mg/dL, and LDL-C ≥ 160 mg/dL were excluded at the time of data analysis.

Anthropometric characteristics and laboratory data are shown in Table 1.

### 2.3. Pulse Wave Velocity: Distances and Algorithms

After blood collection, subjects were taken to the laboratory for noninvasive vascular assessment. Vascular evaluation consisted in measuring complementary structural and functional vascular parameters. Subjects retained for the present analysis (*n* = 429) are a subgroup of the CUiiDARTE Project database [13, 14]. The database includes patients and subjects in whom we evaluated (1) common (CCA), internal and external carotid arteries plaque presence, (2) CCA intima-media thickness and instantaneous diameter waveforms, (3) CCA stiffness (percentual pulsatility, compliance, distensibility, and stiffness index), (4) aortic stiffness (PWV), and (5) peripheral and central (aortic) pressure levels and pulse wave analysis, together with medical history and laboratory analysis. PWV recordings are analyzed in this work. 

**Figure 1:**
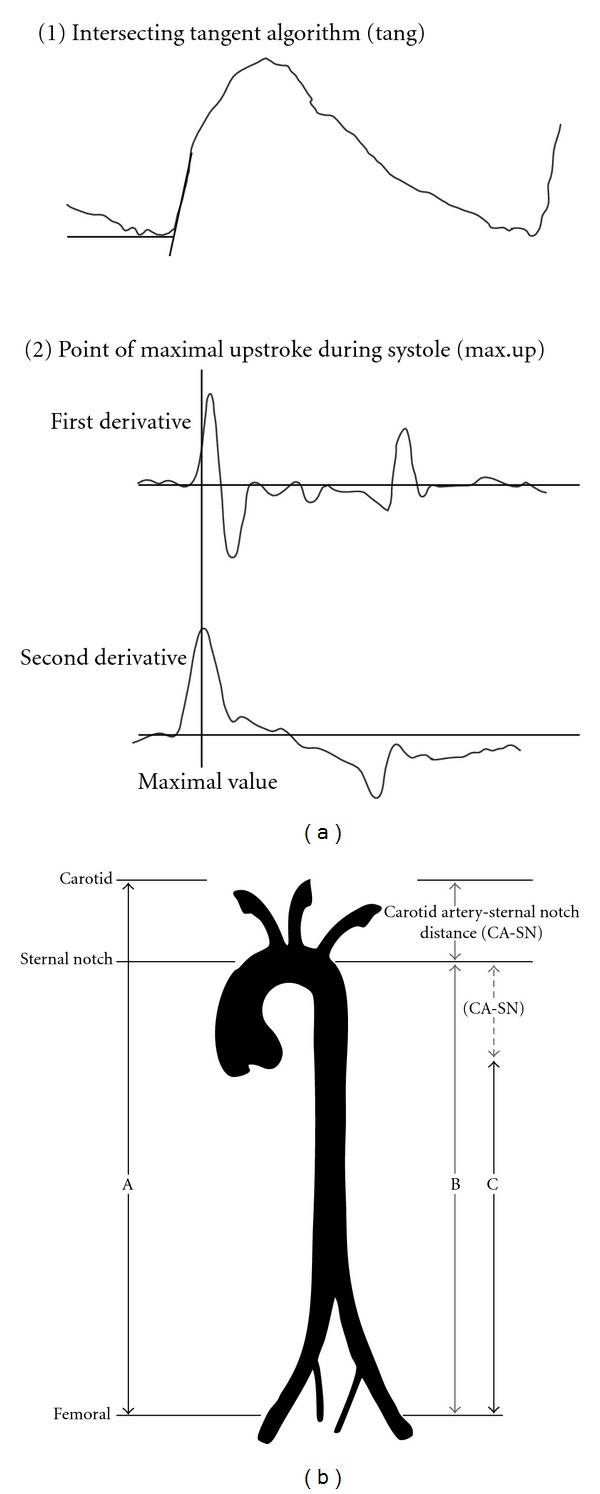
(a) Algorithms employed to determine the carotid-femoral pulse transit time (PTT): intersecting tangent algorithm (tang) and maximal upstroke during systole (max.up). (b) Distances employed to determine the carotid-femoral PWV. Distance A: direct distance between the carotid and femoral region (direct). Distance B: distance between the sternal notch and the femoral region (sn-fem). Distance C: subtracted distance, obtained as B minus the carotid to sternal notch distance (subtracted). Combing the two PTT algorithms and the three distances, six PWV were quantified: PWV_direct/tang_, PWV_direct/max.up_, PWV_sn-fem/tang_, PWV_sn-fem/max.up_, PWV_subtracted/tang_, and PWV_subtracted/max.up_. In addition the real PWV was calculated (see text).

Carotid and femoral pulse waves were recorded using mechanotransducers simultaneously placed on the skin over the carotid and femoral arteries (subjects in supine position) [13, 14]. Once quality pulse waveforms were obtained, digitization was finished and the time delay between the waveforms (pulse transit time) was measured. To this end we considered the two most popular algorithms used to detect the wave foot: the intersecting tangent algorithm (tang) and the point of maximal upstroke during systole (max.up) (Figure 1). Direct, sternal notch-femoral, and subtracted distances were used (Figure 1). Then, six PWV values were obtained: PWV_direct/tang_, PWV_direct/max.up_, PWV_sn-fem/tang_, PWV_sn-fem/max.up_, PWV_subtracted/tang_, and PWV_subtracted/max.up_. The “real” PWV (PWV_Real_) was calculated according to a recently proposed method [3]. PWV_Real_ is a standardized PWV, obtained multiplying by 0.8 the PWV calculated using the direct distance and the intersecting tangent algorithm (to reach more realistic stiffness values) [3]. In all cases, the PWV was automatically calculated as the quotient between the distance and the pulse transit time difference. The reported value was always the average of at least 8 consecutive beats. Brachial pressure and heart rate were quantified (Omron HEM-433INT Oscillometric System; Omron Healthcare Inc., IL, USA). 

#### 2.3.1. Statistical and Group Analysis

Considering our aim, the population characteristics, the prevalence of cardiovascular disease and risk factors in the Uruguayan population, and considering an *α* = 0.05 (C.I. = 95%), the number of subjects included was enough to perform statistical inference about age-related changes in PWV. We used a probability sample strategy (cluster sampling). The “reference value population” was represented by all included subjects; subjects with optimal/normal blood pressures levels represented the “normal value population” [3]. To determine diagnostic thresholds for men and women combined, we rounded the 95th prediction bands. Subjects were categorized according to the age decade (10–19, 20–29, 30–39, 40–49, 50–59, and 60–69 years old) and blood pressure levels (at the time of the study): optimal (<120/80 mmHg), normal (≥120/80 mmHg and <130/85 mmHg), high normal (≥130/85 mmHg and <140/90 mmHg), grade I hypertension (≥140/90 mmHg and <160/100 mmHg), and grade II/III hypertension (≥160/100 mmHg). Differences among groups were tested by means of ANOVA and Bonferroni's post-test. Statistical analyses were done using Statistical Package for the Social Sciences 17.0 for Windows software.

## 3. Results

Table 1 summarizes clinical and hemodynamic characteristics of the studied subjects. As was expected, there was an aging-associated increase in systolic blood pressure (*P* < 0.05). Diastolic pressure showed lesser aging-associated changes, but it decreased beyond 60 years. Heart rate showed a tendency to decrease with age.

**Figure 2:**
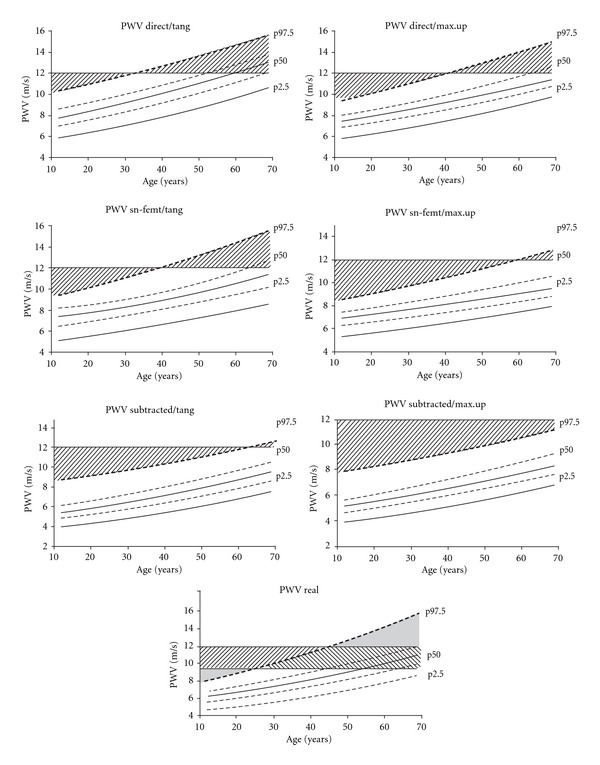
PWV nomograms for the reference population with percentiles (p) 97.5, 50, and 2.5. Broken lines indicate mean confidence interval. The dashed areas illustrate differences between the threshold defined from our population and the fixed unique threshold proposed in the ESH/ESC Guidelines. For PWV_Real_ the fixed unique threshold proposed by Boutouyrie et al. (9.6 m/s) was included [3]. Note that the fixed threshold of PWV = 12 m/s or 9.6 m/s determines an underestimation and overestimation to detect preclinical arterial wall damage in young and old subjects, respectively. Underestimation or overestimation would differ depending on the subjects' age and the algorithm used to assess PWV.

Table 2 shows PWV values (reference levels) for the different age groups considering the algorithms used. Figure 2 shows the PWV nomograms for the whole reference population. There were no gender-related statistical differences in PWV when an isobaric analysis was performed. The expected age-associated increase in PWV was observed in our population (*P* < 0.05) (Table 2 and Figure 2). PWV percentiles differed among the different methods of calculus (*P* < 0.05) (Figure 2 and Table 2). Such differences varied depending on the algorithms and age considered (*P* < 0.05).

In Figure 2, the dashed areas illustrate the differences between the age-related threshold (percentile 97.5) defined from our population and the fixed unique threshold proposed in the ESH/ESC Guidelines (PWV = 12 m/s or 9.6 m/s) [1]. Note that a fixed threshold of PWV = 12 m/s (or 9.6 m/s for PWV_Real_) would underestimate or overestimate arterial wall damage depending on the subjects age. The underestimation or overestimation would differ if the algorithm used to determine the threshold PWV is not considered, and hence the PWV levels are not corrected accordingly.

**Figure 3:**
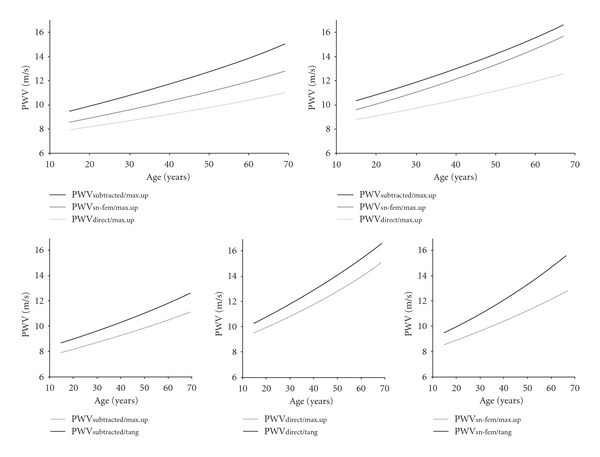
PWV thresholds (percentile 97.5) differences considering different combinations of distances and algorithms used to calculate PWV.

Figure 3 and Table 3 show the percentile 97.5 for PWV obtained using different distances and algorithms. Note that the differences in PWV among the methods of calculus varied depending on the subjects' age (*P* < 0.05). Differences between maximum and minimum values were higher in elderly individuals than in young subjects (*P* < 0.05).

For a given distance, maximal PWV values were obtained with the intersecting tangent (tang) algorithm. Additionally, as was expected, for a given algorithm PWV was higher when the direct distance was considered (Figure 3). The referred differences increased with age.

Table 4 shows PWV levels (normal and reference values) for the whole population and the different methods used, considering age and blood pressure levels. Subjects with optimal or normal blood pressure had the lowest PWV levels (normal population). In general terms, subjects with normal blood pressure had PWV values higher than those of subjects with optimal blood pressure (*P* < 0.05).

## 4. Discussion

The definition of normal and reference values represents a critical step in the implementation of PWV as a clinical tool for detecting subclinical organ damage in routine patient workup. Reference values have been defined for European populations [3]. However, given the population-based differences in the vascular behavior in physiological and pathological conditions, values obtained in a given population may not be applicable to another one. On the other hand, values previously reported were obtained considering a single PWV methodological approach, restricting their applicability to specific measurement protocols or devices. In this work, PWV values were obtained in a Uruguayan population considering different path lengths and algorithm of calculus.

To contribute to overcome limitations in the clinical use of PWV and other vascular parameters, CUiiDARTE Project was developed, with financial support from the National Agency for Research and Innovation (ANII, http://www.anii.org.uy/). The project aimed at building a National Database integrating noninvasive vascular parameters considered markers of subclinical arterial damage [13, 14]. In this work, we obtained and analyzed data from CUiiDARTE to establish normal/reference values for PWV.

*This work main contribution is the establishment of normal/reference PWV values for Uruguayans, obtained in a population*-*based study and considering age, blood pressure levels, and different methodological approaches. Our work has the strength of being the first in Latin America that applies an integrative approach to characterize age-related changes and determine normal/reference PWV values. The obtained data could be used in the vascular diagnosis to define/differentiate normal changes (i.e., due to haemodynamic conditions) and abnormal or disease-related vascular variations. In addition*,* they could aid in the individual cardiovascular risk definition.*

### 4.1. PWV Normal and Reference Values: Algorithm and Path-Length Consideration

As was mentioned, PWV values depend on the algorithm used to detect the so-called “foot of the wave” and the path length considered. In real terms most of the systems used to assess PWV do not detect the “foot of the wave,” but the pulse transit time is determined as the time difference between similar singular points in the carotid and femoral waves (Figure 1). The singular point chosen depends on the wave considered (flow, pressure, or diameter) and the algorithm used. The most used algorithms are the intersecting tangent (i.e., used by the SphygmoCor system) and the point of maximal upstroke during systole (i.e., used by the Complior system).

When applying different algorithms to the same waves we obtained differences in PWV mean values even higher than 20%. The differences varied depending on age, with major differences in elderly subjects. Then, age-related changes in PWV could be influenced by the methods used to assess PWV [17, 18]. To explain the mechanism underlying the described finding was beyond our work aim. However, at least in theory, the algorithm-dependent differences in PWV could be explained by dissimilar effects of wave propagation changes on the singular points detected by the algorithms. For instance, the foot of the wave identified by the intersecting tangent method is least likely to be influenced by the wave distorsion during its propagation. Our finding agrees with Millasseau et al. who described that the differences in PWV attributable to the timing algorithm used varied depending on the stiffness levels [18]. The higher the PWV levels, the higher the differences. Related with this, it is noteworthy that PWV increased with aging.

The evaluation of the meaning of a given PWV value regardless of the algorithm used could lead to mistakes. On the other hand, and related with that stated above if a PWV value is used as a cut-off value to define vascular damage, it is necessary to know the method used to determine it and the corresponding values for other algorithms. Anyway, the use of a single cut-off value has limitations (see below).

When considering the different path lengths at the time of calculating PWV, we found that, for a given algorithm, the longer the path length considered, the higher the PWV. Then, as was stated for the algorithms of calculus, an adequate interpretation of a PWV value requires the knowledge of the distance used in its determination. In addition, when using a cut-off value the distance considered to calculate it must be known.

Our work was not developed to determine which is the best method for measuring PWV (if there is one). To this end, it would be useful to compare the different methodological approaches with a “gold standard” (definitive) method, but there is no consensus about which would be such method. There are works that support the use of a given approach. For instance, it was described that PWV values, measured using magnetic resonance imaging (MRI), were in agreement with absolute PWV values noninvasively obtained using the subtracted distance [5]. Additionally, in a recent invasive study the same distance (subtracted) was the closest to the PWV measured during catheter withdrawal from the ascending aorta to the aortic bifurcation [6]. In addition, compared with invasive studies the direct distance resulted in a PWV overestimation of 2-3 m/s [6]. Then, it was proposed by Weber et al. that for the purpose of standardization and comparability between different noninvasive systems (devices), the method that employs the subtracted distance should be recommended for noninvasive PWV measurement [7]. The topic remains controversial since the use of the direct is proposed by other authors [17]. In our opinion, until there is no consensus as to what constitutes the definitive method to assess PWV, we must be aware of the importance of considering the methodological issues for an adequate interpretation of the PWV evaluation. Tables including normal/reference PWV values for the different methods should be constructed.

### 4.2. PWV Normal and Reference Values: Aging and Blood Pressure Levels Consideration

Aging is associated with an increase in vascular stiffness. Accordingly, when using PWV to evaluate the vascular system (i.e., as target organ damage indicator) the expected changes (increase) in PWV due to normal aging should be known. On the other hand, as it is widely known, the arterial stiffness depends on blood pressure levels. The higher the pressure levels the stiffer the artery (higher PWV). Then, if PWV is to be used as target organ damage marker, the PWV levels explained by haemodynamic conditions should be known. This is not a minor issue since hypertension associates changes in the vascular wall that result in stiffening of the vessel. Establishing PWV reference levels for different blood pressure values would help to identify individuals in whom an increased PWV represents the presence of vascular damage or subjects with PWV values explained by the pressure levels. In this work, and as was previously described by Boutouyrie and Vermeersch [3], we defined PWV normal/reference values as a function of age and blood pressure levels. The contribution of risk factors other than age and blood pressure to PWV is either small or nonsignificant. Then standardizations considering other factors were not necessary [3, 8]. 

The aging-associated changes in PWV and the pressure dependence reinforce the referred limitations of using a single PWV value as cut-off level. About this, as can be seen in Figure 2 if a single cut-off value (i.e., 12 m/s as the European Society of Hypertension suggests) is selected regardless of the subject age, the vascular damage and/or the cardiovascular risk would be underestimated in young subjects and overestimated in old subjects. Beyond 60 years old 50% of the subjects fall at or above the threshold (12 m/s) when PWV_Direct/tang_ method was employed.

## 5. Conclusions

Age-related PWV profiles were obtained in the context of the CUiiDARTE Project for a Uruguayan asymptomatic population. Taking into account the importance of the methodological approach, the subject age, and the pressure levels for an adequate evaluation of the PWV, we defined PWV normal/reference values for our population considering (a) the subjects age, (b) the pressure levels, and (c) the algorithm and (d) the distance used to calculate the PWV.

The work has the strength of being the first in Latin America that applies an integrative approach to characterize age-related changes and normal and reference values of PWV. The obtained data could be used in the vascular diagnosis to define/differentiate normal changes and abnormal or disease-related vascular variations and/or in detecting increased risk of cardiovascular complications.

## Figures and Tables

**Table 1:** Clinical and hemodynamic characteristics.

Group	10–19 years	20–29 years	30–39 years	40–49 years	50–59 years	60–69 years
*N*	61	103	60	71	66	68
Age (years)	15 ± 2	24 ± 2^a^	34 ± 2^ab^	45 ± 3^abc^	54 ± 3^abcd^	64 ± 4^abcde^
Anthropometric measurements						
Body height (m)	165 ± 9	167 ± 9	166 ± 9	165 ± 9	165 ± 8	160 ± 10
Body weight (kg)	57 ± 11	63 ± 12	72 ± 16^ab^	73 ± 16^ab^	71 ± 16^ab^	71 ± 12^ab^
BMI (kg/m^2^)	21 ± 3	23 ± 4^a^	26 ± 3^ab^	26 ± 3^ab^	25 ± 4^ab^	27 ± 2^ab^
Peripheral hemodynamics parameters						
Systolic pressure (mmHg)	118 ± 13	125 ± 12^a^	126 ± 14	130 ± 13^ab^	132 ± 16^abc^	144 ± 19^abcde^
Diastolic pressure (mmHg)	64 ± 10	71 ± 8^a^	74 ± 11^a^	78 ± 9^ab^	79 ± 11^ab^	75 ± 10^ab^
Pulse Pressure (mmHg)	48 ± 6	51 ± 8	53 ± 10	54 ± 8^a^	56 ± 9^a^	69 ± 13^abcde^
Heart rate (bpm)	78 ± 13	73 ± 11	72 ± 8^a^	72 ± 12^a^	73 ± 12^a^	63 ± 10^abcde^
Laboratory						
Total cholesterol (mg/dL)	183.7 ± 32.1	172.3 ± 23.3	189.3 ± 21.4	184.6 ± 17.0	217.0 ± 8.6^abd^	190.4 ± 20.3
HDL-cholesterol (mg/dL)	62.1 ± 7.3	63.4 ± 13.7	66.0 ± 26.6	67.4 ± 6.3	73.2 ± 19.4^ab^	65.2 ± 9.6
LDL-cholesterol (mg/dL)	101.2 ± 21.2	90.1 ± 19.3	109.5 ± 28.5	100.9 ± 5.8	126.9 ± 12.2^abd^	91.2 ± 12.1^a^
Triglycerides (mg/dL)	96.4 ± 47.2	83.2 ± 22.4	56.2 ± 21.0^ab^	58.0 ± 2.0^ab^	60.2 ± 21.3^ab^	64.2 ± 18.0^ab^

Mean value ± SD. *N*: number of subjects, BMI: body mass index, HDL and LDL: high and low density cholesterol, respectively.

Statistics: ^a, b, c, d, e ^: *P* < 0.05 with respect to 10–19, 20–29, 30–39, 40–49, and 50–59 years, respectively (ANOVA + Bonferroni Test).

**Table 2:** PWV levels (reference population).

	All					
	10–19	20–29	30–39	40–49	50–59	60–69
PWV direct/tang						
Mean	7.7	8.8^a^	10.2^a,b^	10.7^a,b^	11.1^a,b^	14.8^a,b,c,d,e^
SD	1.1	1.5	1.4	1.5	1.5	5.8
*P* 25	7.2	7.7	9.1	9.6	10.2	12.5
*P* 50	7.5	8.8	10.4	10.9	10.9	13.2
*P* 75	8.2	9.7	11.3	11.5	11.7	13.7
*P* 90	9.0	10.8	11.7	12.5	13.1	15.1
PWV sn-fem/tang						
Mean	7.7	7.7	8.6	9.5^a,b^	9.9^a,b,c^	13.1^a,b,c,d,e^
SD	1.1	1.4	1.4	1.7	1.6	4.7
*P* 25	7.2	6.6	7.7	8.3	8.7	10.8
*P* 50	7.5	7.6	8.9	9.5	9.5	11.5
*P* 75	8.2	8.5	9.6	10.3	10.3	13.0
*P* 90	9.0	9.5	10.0	11.9	12.6	14.4
PWV subtracted/tang						
Mean	5.3	6.4^a^	7.4^a,b^	8.0^a,b^	8.3^a,b^	10.7^a,b,c,d^
SD	1.2	1.4	1.3	1.6	1.5	4.0
*P* 25	4.9	5.4	6.2	6.8	7.2	8.9
*P* 50	5.1	6.3	7.3	7.8	7.9	9.5
*P* 75	5.6	7.3	8.2	9.0	8.7	10.3
*P* 90	6.3	8.1	8.7	9.9	10.7	11.1
PWV direct/max.up						
Mean	7.3	8.3^a^	9.3^a,b^	9.8^a,b^	10.2^a,b,c^	12.4^a,b,c,d,e^
SD	0.9	1.2	1.1	1.3	1.4	2.7
*P* 25	6.9	7.4	8.3	8.9	9.3	10.9
*P* 50	7.2	8.3	9.3	9.8	9.8	11.4
*P* 75	7.7	9.1	10.1	10.3	11.0	12.4
*P* 90	8.5	9.9	10.5	11.5	12.4	14.4
PWV sn-fem/max.up						
Mean	7.2	7.1	7.9^b^	8.4^a,b^	8.8^a,b,c^	10.6^a,b,c,d,e^
SD	0.9	1.1	0.9	1.2	1.4	2.4
*P* 25	6.9	6.2	7.0	7.5	7.9	9.3
*P* 50	7.2	7.1	7.9	8.4	8.5	9.7
*P* 75	7.6	7.9	8.7	9.0	9.5	10.8
*P* 90	8.1	8.6	8.9	10.0	10.7	12.3
PWV subtracted/max.up						
Mean	5.0	6.0^a^	6.7^a^	7.1^a,b^	7.3^a,b^	8.8^a,b,c,d^
SD	1.0	1.1	1.1	1.2	1.4	2.0
*P* 25	4.6	5.1	5.9	6.2	6.6	7.7
*P* 50	4.9	5.9	6.7	6.8	7.0	8.2
*P* 75	5.2	6.6	7.3	7.8	7.7	9.1
*P* 90	5.9	7.3	7.6	8.5	9.0	10.2
PWV real						
Mean	6.1	7.2^a^	8.2^a,b^	8.9^a,b^	9.4^a,b,c^	12.5^a,b,c,d,e^
SD	0.9	1.3	1.2	1.6	1.8	4.3
*P* 25	5.7	6.2	7.3	7.8	8.2	10.3
*P* 50	6.0	7.1	8.4	8.9	8.9	10.8
*P* 75	6.5	7.9	9.0	9.5	10.1	12.1
*P* 90	7.2	9.0	9.6	11.1	12.2	15.1

Statistics: ^a, b, c, d, e ^, *P* < 0.05 with respect to 10–19, 20–29, 30–39, 40–49, and 50–50 years, respectively (ANOVA + Bonferroni).

**Table 3:** PWV thresholds (reference population).

	≤19 y.	20–29 y.	30–39 y.	40–49 y.	50–59 y.	60–69 y.
PWV direct/max.up	9.1	10.9	11.0	12.3	13.7	14.7
PWV direct/tang	9.8	11.7	12.5	13.5	14.2	16.5
PWV sn-fem/max.up	8.9	9.1	9.4	10.7	12.2	12.6
PWV sn-fem/tang	9.8	10.5	10.6	12.6	13.7	16.0
PWV subtracted/max.up	7.6	8.7	8.8	10.0	10.4	10.6
PWV subtracted/tang	8.2	9.5	10.1	11.4	11.4	12.0
PWV real	7.9	10.0	10.1	11.8	13.6	15.6

Statistics: regardless the PWV calculus, thresholds differed (*P* < 0.05) among the different age groups.

**Table 4:** PMV levels considering age and blood pressure.

	Blood pressure category			
	Optimal	Normal	High normal	Grades I/II HTA
PWV direct/max.up				
10–19 years	6.9 (5.8–7.9)	7.4 (5.2–9.6)^#^	7.8 (5.9–9.8)^#&^	(—)
20–29 years	8.0 (5.6–10.4)^a^	8.1 (5.7–10.6)^a#^	8.7 (6.8–10.7)^a#&^	9.1 (6.1–12.0)^#&%^
30–39 years	8.4 (7.7–9.2)^ab^	8.7 (5.6–10.5)^ab#^	9.7(8.9–10.6)^ab#&^	10.4 (9.6–11.2)^b#&%^
40–49 years	8.9 (7.2–10.5)^abc^	9.6 (8.8–10.0)^abc#^	10.0 (7.6–12.5)^abc#&^	10.6 (5.6–15.5)^b#&%^
50–59 years	9.7 (8.0–11.4)^abcd^	9.9 (7.8–12.0)^abcd#^	10.4 (6.9–13.9)^abcd#&^	10.5 (7.6–13.4)^b#&^
PWV direct/tang				
10–19 years	7.2 (5.9–8.5)	7.9 (5.0–10.7)^#^	8.2 (6.3–10.1)^#&^	(—)
20–29 years	8.6 (5.5–11.7)^a^	8.7 (5.5–11.9)^a#^	9.4 (6.8–12.1)^a#&^	10.0 (5.9–14.1)^#&%^
30–39 years	9.6 (7.6–11.5)^ab^	9.7 (3.5–15.9)^ab#^	10.8 (9.6–12.0)^ab#&^	11.8 (10.5–13.1)^b#&%^
40–49 years	9.8 (7.4–12.1)^ab^	10.9 (9.6–12.2)^abc#^	11.5 (8.0–15.0)^abc#&^	12.5 (7.2–17.9)^bc#&%^
50–59 years	10.7 (8.4–13.0) ^abcd^	11.1 (8.9–13.3)^abc#^	11.6 (8.4–14.9)^abc#&^	13.0 (8.2–17.2)^bcd#&%^
PWV sn-fem/max.up				
10–19 years	6.8 (5.8–7.9)	7.2 (5.1–9.3)^#^	7.4 (6.2–8.6)^#&^	(—)
20–29 years	6.9 (4.8–9.0)	7.2 (5.4–8.9)^#^	7.6 (6.0–9.2)^a#&^	7.9 (5.2–10.6)^#&%^
30–39 years	7.2 (6.4–8.0)^ab^	7.5 (3.6–11.3)^ab#^	8.3 (7.5–9.2)^ab#&^	8.8 (8.2–9.5)^b#&%^
40–49 years	7.6 (5.8–9.5)^abc^	8.3 (7.5–9.2)^abc#^	8.6 (6.4–10.7)^abc#&^	9.3 (4.4–14.1)^bc#&%^
50–59 years	8.3 (6.9–9.8)^abcd^	8.5 (6.5–10.4)^abc#^	8.9 (5.8–12.0)^abcd#&^	9.6 (7.6–11.5)^bcd#&%^
PWV sn-fem/tang				
10–19 years	7.2 (5.9–8.5)	7.6 (5.0–10.3)^#^	7.8 (6.1–9.6 )^#&^	(—)
20–29 years	7.4 (4.6–10.2)^a^	7.6 (5.1–10.1)^#^	8.1 (5.7–10.4)^a#&^	8.7 (5.0–12.4)^#&%^
30–39 years	7.9 (5.7–10.2)^ab^	8.7 (7.4–12.8)^ab#^	9.2 (8.1–10.4)^ab#&^	10.1 (9.0–11.2)^b#&%^
40–49 years	8.4 (6.0–10.8)^abc^	9.4 (8.3–10.5)^abc#^	9.8 (6.7–12.8)^abc#&^	10.7 (3.9–17.5)^bc#&%^
50–59 years	9.3 (7.2–11.5)^abcd^	9.6 (6.8–12.5)^abcd#^	10.2 (5.6–14.8)^abcd#&^	11.1 (7.6–14.6)^bcd#&%^
PWV subtracted/max.up				
10–19 years	4.6 (3.8–5.4)	4.8 (3.4–6.3)	5.0 (4.2–5.8)^#&^	(—)
20–29 years	5.7 (3.7–7.7)^a^	5.9 (3.2–8.5)^a^	6.3 (4.0–8.6)^a#&^	6.5 (4.0–9.0)^#&%^
30–39 years	6.1 (4.9–7.4)^ab^	6.2 (2.8–9.6)^ab^	6.9 (6.1–7.8)^ab#&^	7.3 (5.4–10.3)^b#&%^
40–49 years	6.1 (5.0–7.2)^ab^	7.0 (5.9–8.2)^abc#^	7.3 ( 5.6–9.0 )^abc#&^	7.7 (3.5–12.0)^bc#&%^
50–59 years	6.9 (5.7–8.2)^abcd^	7.0 (5.3–8.8 )^abc^	7.4 (4.7–10.2)^abc#&^	8.0 (5.9–9.6)^bcd #&%^
PWV subtracted/tang				
10–19 years	4.8 (3.9–5.8)	5.1 (3.4–6.9)^#^	5.4 (4.3–6.4)^#&^	(—)
20–29 years	6.3 (3.5–9.0)^a^	6.3 (3.0–9.6)^a^	6.8 (3.9–9.7)^a#&^	7.1 (3.8– 10.4)^#&%^
30–39 years	6.7 (5.2–8.2)^ab^	6.9 (2.2–11.6)^ab^	7.7 (6.6–8.7)^ab#&^	8.4 (7.6–9.3)^b#&%^
40–49 years	6.8 (5.2–8.4)^ab^	7.7 (6.8–8.7)^abc#^	8.2 (5.7–10.7)^abc#&^	8.9 (3.0–14.8)^bc#&%^
50–59 years	7.8 (6.0–9.5)^abcd^	8.0 (5.5–10.5)^abcd#^	8.5 (4.5–12.4)^abcd#&^	8.8 (5.4–12.2)^bc#&%^
PWV real				
10–19 years	5.8 (4.7–6.8)	6.3 (4.0–8.5 )^#^	6.3 (4.8–7.7)	(—)
20–29 years	6.9 (4.4–9.4)^a^	7.0 (4.4–9.5)^a^	7.6 (5.4–9.7)^a#&^	8.0 (4.7–11.2)^#&%^
30–39 years	7.7 (6.1–9.2)^ab^	7.8 (2.8–12.7)^ab^	8.6 (7.7–9.6)^ab#&^	9.5 (8.4–10.5 )^b#&%^
40–49 years	7.8 (6.0–9.7)^ab^	8.7 (7.5–10.1)^abc#^	9.2 (6.4–12.0)^abc#&^	9.9 (7.9–15.7)^bc#&%^
50–59 years	8.9 (6.4–11.5)^abcd^	9.0 ( 7.6–11.0)^abcd#^	9.5 (5.3–13.7)^abcd#&^	10.4 (6.9–13.7)^bcd#&%^

Statistics: ^a, b, c, d, e ^, *P* < 0.05 with respect to 10–19, 20–29, 30–39, 40–49, and 50–59 years, respectively (ANOVA + Bonferroni).

^#, &, %^, *P* < 0.05 with respect to optimal, normal, and high normal blood pressure level, respectively (ANOVA + Bonferroni).
